# Impact of enhanced recovery after cesarean protocol on postoperative urinary retention

**DOI:** 10.1007/s11255-025-04610-2

**Published:** 2025-06-26

**Authors:** M. Kaitlyn Coghlan, Liviu Cojocaru, Suzanne Alton, Autusa Pahlavan, Ariel Trilling, Hyunuk Seung, Bhavani Kodali, Sarah Crimmins, Katherine Goetzinger

**Affiliations:** 1 Department of Obstetrics, Gynecology, and Reproductive Sciences, University of Maryland School of Medicine, Baltimore, MD USA; 2 Division of Maternal-Fetal Medicine, Department of Obstetrics and Gynecology, University of Miami Miller School of Medicine, Miami, FL USA; 3 Department of Obstetrics and Gynecology, University of Rochester Medical Center, Rochester, NY USA

**Keywords:** Postoperative urinary retention, Postoperative complications, Cesarean delivery, Enhanced recovery, Recovery protocols, ERAS

## Abstract

**Objective:**

Postoperative urinary retention (POUR) remains a common concern associated with cesarean delivery (CD). Recently, efforts have been made to expedite recovery after CD through Enhanced Recovery After Cesarean (ERAC) pathways. We aimed to evaluate whether the implementation of an ERAC protocol impacts the incidence of POUR after CD.

**Methods:**

This is a secondary analysis of a prospective, longitudinal, quality improvement (QI) study of patients undergoing CD before and after implementation of an ERAC protocol. CD patients requiring general anesthesia, significantly complicated CD, and patients with chronic pain disorders were excluded. The primary outcome was POUR, defined as failure of spontaneous voiding 6 hours (h) after Foley catheter removal. Secondary outcomes were the duration of Foley catheter placement and replacement of Foley catheter.

**Results:**

Three hundred-eight patients were included. The incidence of POUR was similar between groups pre-ERAC (44, 22.6%) vs post-ERAC (36, 31.8%), *p* = 0.08. Time to Foley removal following CD was significantly decreased in the post-ERAC cohort [10.1 h (7.2, 13.7) vs. 12.5 h (10.9, 17.8)] (*p* < 0.001). Need for Foley catheter replacement occurred at a similar rate (pre-ERAC: 1.0%; post-ERAC: 0.9%, *p* = 0.7).

**Conclusions:**

There was no significant difference in POUR after implementing an ERAC protocol, although a trend toward increased incidence was noted. Post-ERAC implementation resulted in a significant decrease in the duration of Foley catheter placement.

## Introduction

Level I evidence supports enhanced recovery after surgery protocols being associated with improved perioperative outcomes and should be considered standard of care at the time of gynecologic surgery [1–3]. Similarly, cesarean delivery (CD), one of the most common surgical procedures performed in the United States [4], has seen the development of enhanced recovery after cesarean delivery (ERAC). These multidisciplinary, standardized care approaches aim to improve outcomes surrounding CD [5]. Much of the focus of these pathways has been on decreasing inpatient and outpatient opioid use, as well as decreasing the length of inpatient stay (LOS), through the implementation of multimodal analgesia, improved patient education, and early ambulation [6–9]. While beneficial, the impact of measures taken to achieve these goals on other pelvic floor perioperative outcomes is not known.

Postpartum urinary retention remains a common concern associated with childbirth. Postoperative urinary retention (POUR) following cesarean delivery is not well studied but may occur in up to 25% of patients [10]. The pathophysiology of urinary retention in the postpartum setting is likely multifactorial with physiologic, neurologic, and mechanical factors contributing. Urinary retention in the postpartum setting was historically thought to be transient, but evidence regarding long-term adverse effects is lacking [11]. Because the long-term sequelae of urinary retention in the postpartum setting are not known or well studied, screening for elevated post-void residual volume (PVR) is not routine as is often the case in gynecologic/urogynecologic surgery [12, 13]. While long-term problems with micturition are an established risk in patients with recurrent urinary retention, persistent voiding dysfunction has also been described after a single episode of bladder overdistension [14–16].

Enhanced recovery protocols decrease the physiologic stress of surgery and are associated with improved perioperative outcomes [1]. In an effort to shorten the time to ambulation, an ERAC protocol in our institution established the early removal of Foley catheters following CD. We aimed to evaluate if the implementation of an ERAC protocol impacts the incidence of POUR in patients undergoing CD.

## Methods

This is an Institutional Review Board-approved secondary analysis of a prospective, longitudinal, quality improvement (QI) study of all patients 18 years of age and older undergoing uncomplicated CD at a single, urban tertiary referral center. The parent study compared outcomes in patients before (pre-ERAC) and after (post-ERAC) implementation of the ERAC algorithm [9]. Patient enrollment pre-ERAC occurred from October 2019 until February 2020. Interventions were designed following Enhanced Recovery After Surgery (ERAS) Society guidelines for CD [6–8]. In March of 2020, the ERAC algorithm was implemented on our Obstetric Care Unit (OBCU). A “wash-out period” of 2 months was allowed to permit staff adjustment and implementation of this new protocol. Following this period, post-ERAC enrollment occurred from May 2020 until September 2020.

Exclusion criteria were dictated by the parent study and included CD completed under general anesthesia, those complicated by massive transfusion events (defined as transfusion of six or more units of packed red blood cells) or bowel injury, CD requiring recovery in the intensive care unit, and skin incision other than Pfannenstiel, as these factors may influence postoperative opioid use and recovery. Additionally, to further reduce factors that may result in increased opioid use, patients with chronic pain disorders, chronic opioid use, acute postpartum depression, or patients whose neonate demised before their discharge were also excluded.

Current practice was reviewed, and the following changes were implemented: multimodal analgesia [post-ERAC: Acetaminophen 1 g PO and Gabapentin 600 mg PO once preoperatively; Acetaminophen 1 g PO q6h, Ibuprofen 800 mg q8h, and Gabapentin 300 mg q8h scheduled postoperatively; Oxycodone 2.5–5 mg q4h or Tramadol 25–50 mg q4h as needed (PRN) for patients not on a patient-controlled analgesia (PCA) pump postoperatively; pre-ERAC: Acetaminophen 1 g per rectum intraoperatively; Acetaminophen 1 g PO q6h PRN, Ibuprofen 800 mg q8h PRN, and Oxycodone 5–10 mg q4h PRN or Tramadol 25–50 mg q4h PRN postoperatively], reduction of preoperative fasting time (6 h for light meal and 2 h for intake of clear liquids), standardization of goals for early ambulation, and patient education. To facilitate decreased time to ambulation, intravenous (IV) fluids were discontinued in a standardized manner, and urinary catheter removal was expedited. Peripheral IV fluids were discontinued once 600 mL of oral intake was tolerated, or at 8:00 AM for patients delivered after 8:00 PM (if tolerating oral intake).

Urinary catheter was recommended for removal at 6 h postoperatively or 2 h after ambulation, whichever came first, and was removed no later than 12 h after CD. For patients on magnesium sulfate, the intravesical catheter was maintained at the provider’s discretion. Prior to implementation of ERAC, the timing of removal of Foley catheter was not standardized and typically occurred 12–24 h postoperatively. Patients were not routinely actively managed by nursing or support staff with timed attempted voids while awaiting a spontaneous void before or after ERAC implementation. Voided volumes were recorded, but no specific parameters were set for voiding adequacy. Screening for elevated PVR was also not routine postpartum practice, as such, POUR was defined as failure of spontaneous voiding trial (or inability to void spontaneously) 6 h after Foley catheter removal [17, 18]. Management of POUR was not standardized but, in retrospective chart review, was most often found to include straight catheterization of patients once POUR was recognized, followed by reinsertion of Foley catheter if POUR persisted after an additional 6 h had passed (12 h after removal of Foley catheter placed for CD). The timing of these events was documented by nursing staff in the electronic medical record in a standardized manner implemented with the ERAC protocol.

The primary outcome of this study was POUR, defined as failure of spontaneous voiding trial 6 h after Foley catheter removal. Secondary outcomes were the duration of Foley catheter placement and the need for replacement of Foley catheter.

Descriptive statistics were used for incidence, median, and mean. Baseline demographics and outcomes were compared between pre-ERAC and post-ERAC cohorts using *χ*^2^ test or Fisher’s exact test for categorical variables and the Wilcoxon rank-sum test or Student’s *t* test for continuous variables. Normality of distribution was established using the Kolmogorov–Smirnoff test. A *p* value of < 0.05 was considered statistically significant.

## Results

Three hundred and eight (*n* = 308) patients undergoing CD were enrolled in the study, 196 in the pre-ERAC cohort and 112 in the post-ERAC cohort. Baseline characteristics were comparable with regard to age, race and ethnicity, gravity, parity, and most preexisting medical conditions (Table 1). Race and ethnicity were assigned based on that designated by patients during hospital registration. The incidence of diabetes mellitus was higher in the pre-ERAC cohort (12.8 vs. 3.6%, *p* = 0.008). Higher BMI was noted in the post-ERAC cohort [35.8 (30.9–41) vs. 32.1 (28.3–40.2), *p* < 0.001] (Table 1). The rate of history of prior laparotomy other than CD was higher in the post-ERAC cohort (9.8% vs. 4.1%, *p* = 0.04). The indications for CD were overall similar, with the exception that patients in the post-ERAC cohort had a lower incidence of delivery for placental abnormality (0.9% vs. 6.1%, *p* = 0.04).

**Table 1:** Demographic and medical characteristics

	Total (*n* = 308)	preERAC (*n* = 196)	postERAC (*n* = 112)	*p* value
Age, mean (SD)	30.6 (6.1)	30.6 (6.4)	30.7 (5.5)	0.9
Race, *n* (%)				0.2
Hispanic	17 (5.5)	7 (3.6)	10 (8.9)	
Non-Hispanic Black	195 (63.3)	121 (61.7)	74 (66.1)	
Non-Hispanic White	81 (26.3)	56 (28.6)	25 (22.3)	
Asian	10 (3.3)	8 (4.1)	2 (1.8)	
Other	5 (1.6)	4 (2.0)	1 (0.9)	
BMI, median (IQR)	33.3 (28.3, 40.2)	32.1 (27.5, 39.5)	35.8 (30.9, 41)	0.0002
Gravida, median (IQR)	3 (2, 4)	3 (2, 4)	3 (2, 4)	0.3
Para, median (IQR)	1 (0, 2)	1 (0, 2)	1 (0, 1.5)	0.1
Aborta, median (IQR)	1 (0, 2)	1 (0, 2)	0.5 (0, 1)	0.6
Medical problems				
DM, *n* (%)	29 (9.4)	25 (12.8)	4 (3.6)	0.008
HTN, *n* (%)	61 (19.8)	36 (18.4)	25 (22.3)	0.4
Asthma, *n* (%)	67 (21.8)	42 (21.4)	25 (22.3)	0.9
CKD, *n* (%)	2 (0.7)	2 (1.0)	0	0.5
PIH, *n* (%)	61 (19.8)	45 (23.0)	16 (14.3)	0.07
GDM, *n* (%)	20 (6.5)	7 (3.6)	13 (11.6)	0.006
Cardiovascular disease, *n* (%)	7 (2.3)	4 (2.0)	3 (2.7)	0.7
Autoimmune, *n* (%)	4 (1.3)	2 (1.0)	2 (1.8)	0.6
Neurologic disease, *n* (%)	12 (3.9)	6 (3.1)	6 (5.4)	0.4
Thyroid disorder, *n* (%)	5 (1.6)	3 (1.5)	2 (1.8)	1.0
MDD, *n* (%)	69 (22.5)	48 (24.5)	21 (18.9)	0.3
On medications, *n* (%)	22 (31.9)	17 (35.4)	5 (23.8)	0.3
Positive toxicology, *n* (%)	31 (10.1)	18 (9.2)	13 (11.6)	0.5
Other medical conditions, *n* (%)	17 (5.5)	9 (4.6)	8 (7.1)	0.3

*ERAC* enhanced recovery after cesarean, *BMI* body mass index, *DM* diabetes mellitus, *HTN* hypertension, *GDM* gestational diabetes mellitus, *MDD* major depressive disorder, *SD* standard deviation, *IQR* interquartile range, *n* number

Time to indwelling urinary catheter removal following CD, reported as median and interquartile range, was significantly decreased in the post-ERAC cohort [10.1 h (7.2, 13.7)] compared to the pre-ERAC cohort [12.5 h (10.9, 17.8)] (*p* < 0.001). Although not statistically significant, POUR incidence was higher post-ERAC (pre-ERAC: 22.6%; post-ERAC: 31.8%, *p* = 0.08). Time to first void following removal of Foley catheter was significantly increased in the post-ERAC cohort, compared to the pre-ERAC cohort [5.0 h (3.4–6.8)] vs [4.0 (2.6–5.9)] (*p* = 0.002), respectively. Need for Foley catheter replacement occurred at a similar rate between both groups (pre-ERAC: 1.0%; post-ERAC: 0.9%, *p* = 0.7) (Table 2). There was no significant difference in time to indwelling catheter removal, incidence of POUR, time to first void, and need for Foley catheter replacement when stratified for Magnesium Sulfate therapy.

**Table 2:** Distribution of lower urinary tract outcomes

	Total (*n* = 308)	preERAC (*n* = 196)	postERAC (*n* = 112)	*p* value
Urinary retention, *n* (%)	84 (27.2)	44 (22.6)	36 (31.8)	0.08
Foley duration, h (IQR)		12.5 (10.9–17.8)	10.1 (7.2–13.7)	0.001
Time to first void, h (IQR)		4.0 (2.6–5.9)	5.0 (3.4–6.8)	0.002
Need for Foley replacement, *n* (%)	3 (0.95)	2 (1. 0)	1 (0.9)	0.7

*ERAC* enhanced recovery after cesarean, *IQR* interquartile range, *n* number

## Discussion

Implementation of an ERAC protocol did not result in a statistically significant difference in rates of POUR following CD. However, there was a trend toward increased incidence of POUR in patients who underwent CD post-ERAC (pre-ERAC: 22.6%; post-ERAC: 31.8%, *p* = 0.08). While not statistically significant, this trend may be clinically meaningful, leading to bladder overdistension and/or elevated PVR that is likely underdiagnosed in postpartum patients, whose care is focused on physical, social, and psychological well-being [19], with the potential for persistent voiding dysfunction following delivery [14–16].

One explanation for the trend toward an increasing incidence of POUR after earlier removal of Foley catheter post-ERAC may be medication effect. Gabapentin was added to the preoperative and postoperative pain control regimen with the implementation of ERAC, and its use has been associated with urinary retention [20], as well as increased odds of urinary retention following mid-urethral sling placement [21]. This is supported by a statistically significant increase in time to first void following removal of the Foley catheter in patients in the post-ERAC cohort. Furthermore, administration of neuraxial morphine with regional anesthesia for postoperative pain control is common in obstetric anesthesia practice and has been associated with increased rates of POUR following CD when compared to the other methods [22]. Use of neuraxial morphine is standard in all patients undergoing cesarean delivery in our institution, except for patients with chronic opioid use to prevent dose-dependent adverse reactions. Administration of anesthetics is documented separately from the electronic medical record used for the other aspects of patient care in our institution, and, thus, compliance with rates and dosing of neuraxial and epidural morphine administration were not studied; however, collaboration between the obstetric and anesthesiology teams in our institution occurred regularly before and during implementation of ERAC, and adherence to standard low–medium doses of neuraxial morphine were noted. Given the benefits of gabapentin and neuraxial analgesia in reduction of pain scores, as well as inpatient and outpatient opioid use in this patient population [9], further study regarding the optimal duration of Foley catheter placement in patients receiving these medications is warranted.

This trend toward increasing incidence of POUR following implementation of ERAC did not result in an increased need for replacement of Foley catheters during the acute postoperative period. Long-term outcomes, however, have not been studied, and follow-up of patient symptoms after recovery from CD is an area for future research. The duration of Foley catheter was shorter following the implementation of ERAC, and this is a function of the study design, but remains an important finding. Early removal of Foley catheters in postpartum patients may increase patient satisfaction and feelings of independence, decrease rates of catheter-associated urinary tract infection (CAUTI), and may facilitate earlier ambulation and shorter hospital LOS. The decision regarding the duration of Foley catheter placement requires careful consideration of the balance between its benefits, and the potential risk of POUR following CD. Further research is needed to better understand the optimal duration of Foley catheter placement, particularly in minimizing the risk of postoperative urinary retention while maximizing patient outcomes.

Strengths of our study include a relatively large sample size of 308 patients from a single, urban, tertiary care center. Inclusion of all patients undergoing uncomplicated CD and undergoing the same ERAC protocol during study enrollment improves generalizability. In addition, a 2 months “wash out” period between evaluation or pre-ERAC and post-ERAC outcomes was performed. This allowed physicians and nursing staff to become familiar with protocols and identify potential pitfalls of implementation.

Our study is not without limitations. This study included women from a single urban tertiary referral center and may not be generalizable to patients undergoing CD in other settings. Due to the retrospective nature of this study as a secondary analysis, the possibility of type 2 error exists. The timing of removal of Foley catheter was not standardized, and, while recommended at 6 h following CD, the mean time to removal of urinary catheter after implementation of ERAC of 10.1 h demonstrates that management varied in clinical practice. Moreover, no specific management protocol for POUR, when diagnosed, was established. Most patients underwent intermittent catheterization to relieve retained volume if POUR was noted, with reinsertion of Foley catheter if persistent POUR was noted after 12 h, but this was not standardized, and our study lacks objective data on elevated PVR, as the volume was not always recorded. Differences in clinical practice as they relate to the management of POUR could affect outcomes. A prospective study comparing outcomes in patients whose Foley catheters were maintained for standardized and, potentially, longer durations with a protocol for the management of POUR when recognized is warranted based on our findings of a trend toward increasing incidence of POUR in patients who underwent CD post-ERAC. Additionally, the association of Gabapentin and types and dosing of regional anesthetics in obstetric populations during CD as they relate to POUR should be studied. Furthermore, our study population includes both patients undergoing scheduled CD, as well as those who underwent CD in labor; it is likely that return to functional voiding patterns differs significantly between these two groups and future studies should stratify accordingly. Given these factors, in addition to alterations in bladder storage and emptying intra- and postpartum, patients may not benefit from a “one size fits all” approach to the removal of Foley catheters postoperatively following CD.

Implementation of an ERAC protocol in our institution improved several metrics of recovery following CD, such as earlier Foley catheter removal, which may contribute to improved recovery and patient comfort; however, ERAC implementation was associated with a trend toward increasing the incidence of POUR. Further evaluation of the impact of an ERAC protocol on POUR and increased partnership between obstetric and urogynecologic providers in the management of POUR in the postpartum period may improve patient outcomes.

## Data Availability

No datasets were generated or analysed during the current study.
